# One-Step Posterior and Anterior Combined Approach for L5 Retroperitoneal Schwannoma Eroding a Lumbar Vertebra

**DOI:** 10.1155/2016/1876765

**Published:** 2016-09-27

**Authors:** Giancarlo D'Andrea, Giovanni Sessa, Veronica Picotti, Antonino Raco

**Affiliations:** S. Andrea Hospital, Institute of Neurosurgery, University of Rome “La Sapienza”, Rome, Italy

## Abstract

We report the case of a large lumbar schwannoma eroding the vertebra and originating from spinal canal with invasion of the retroperitoneal space. We also review all the cases in literature reporting lumbar schwannomas eroding the vertebral bodies and invading the retroperitoneal space focusing on the surgical strategies to manage them. Spinal CT-scan revealed a 44 mm × 55 mm inhomogeneous soft-tissue mass arising from the right L5-S1 neural foramen and its most anterior portion had a clear colliquative aspect. Magnetic resonance image showed a neoplastic lesion with homogeneous low signal in T1WI, heterogeneous signal in T2WI, and strong enhancement in postgadolinium examination. It developed as well in the retroperitoneal space, posteriorly to the iliac vein, up to the psoas muscle with wide erosion of the omolateral conjugate foramen. We performed a one-step combined approach together with the vascular surgeon because the lesion was too huge to allow a complete resection via a posterior approach and furthermore its tight relationship with the psoas muscle and the iliac vessels in the retroperitoneal space should be more safely managed via a retroperitoneal approach. We strongly suggest a 1-step surgery first approaching the dumbbell and the intraspinal schwannomas posteriorly achieving the decompression of the spinal canal and the cleavage of the tumor cutting the root of origin and the vascular supply and valuating the stability of the spine for potential artrodesis procedure. The patient must be then operated on via a retroperitoneal approach achieving the complete en bloc resection of the tumor.

## 1. Introduction

Lumbar schwannoma is a common lesion affecting the peripheral nervous system moreover in the spinal canal or into the vertebral foramina.

Giant lumbar schwannomas have been rarely described in literature [1, 2] and particularly affect the retroperitoneal space; furthermore, intraosseous vertebral schwannomas are also rarer accounting for less than 0,2% of bone tumors [3, 4] while retroperitoneal schwannomas have an incidence between 0,7 and 2,7% [5].

The goal of surgery is the complete removal of these lesions to avoid recurrence but giant retroperitoneal schwannomas present more difficulties to choose the best surgical route and to manage the close relationship with vital abdominal structures.

Furthermore, the erosion of a lumbar vertebra also complicates radical surgery for its challenging approach because of the potential following instability and the inherent literature is quite exiguous.

We report the case of a large lumbar schwannoma eroding the vertebra and originating from spinal canal with invasion of the retroperitoneal space through the neural foramen.

Such lesion involved the psoas muscle and the abdominal vessels requiring first a posterior approach and then a retroperitoneal route together with the vascular surgeon.

We also review all the cases in literature reporting lumbar schwannomas eroding the vertebral bodies and invading the retroperitoneal space focusing on the surgical strategies to manage them.

## 2. Case Report

A 64-year-old male came to our observation complaining of pain localized in the posterior side of his right inferior limb since at least 2 years. Over the last 6 months, he progressively developed numbness in the same dermatome. There was neither obvious relief nor precipitating factors.

At the admission, the neurological examination was unremarkable. Physical examination revealed no palpable mass within the abdomen.

**Figure 1:**
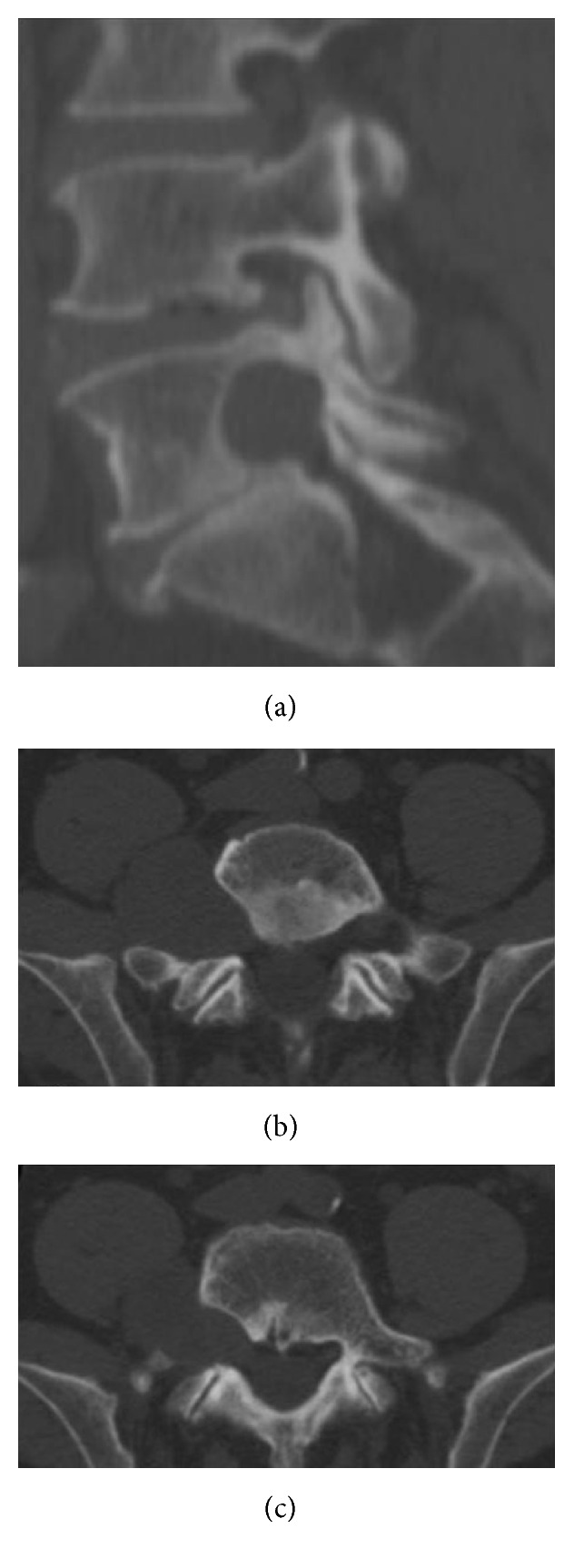
Preoperative spinal CT scan without contrast. The mass widened the omolateral conjugate foramen (a) and extended to the sacrum and to the ipsilateral sacroiliac joint eroding the right posterolateral portion of the L5 vertebral body (b, c).

Spinal CT scan without contrast medium (Figure 1) revealed a 44 mm × 55 mm inhomogeneous soft-tissue mass arising from the right L5-S1 neural foramen and its most anterior portion had a clear colliquative aspect. The mass extended along the lumbar column up to the sacrum and to the ipsilateral sacroiliac joint and eroded the right posterolateral portion of the L5 vertebral body. It developed as well in the retroperitoneal space, posteriorly to the iliac vein, up to the psoas muscle with wide erosion of the omolateral conjugate foramen.

**Figure 2:**
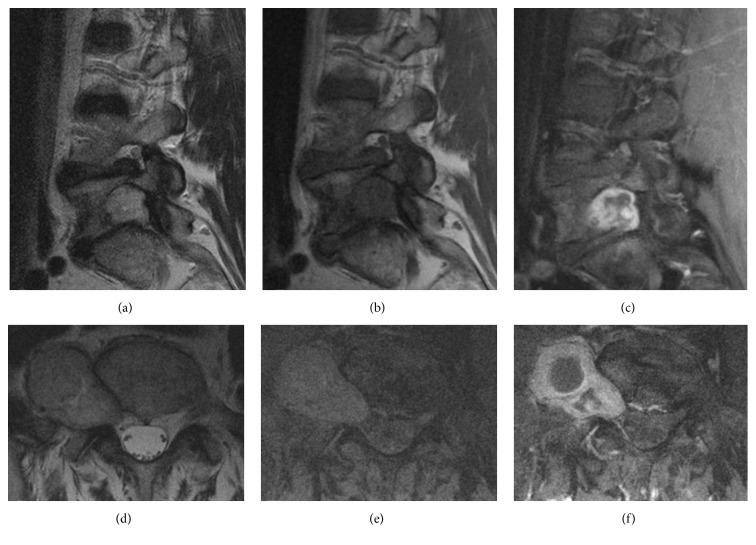
Preoperative spinal MRI. The lesion showed heterogeneous signal in T2WI (a, d), homogeneous low signal in T1WI (b, e, and f), and strong enhancement in postgadolinium examination (c, f). Displacement of the right psoas muscle can be noted (d, e, and f).

Magnetic resonance image (MRI) showed (Figure 2) a neoplastic lesion with homogeneous low signal in T1WI, heterogeneous signal in T2WI, and strong enhancement in postgadolinium examination. The mass extended along the right side of the L5-S1 intervertebral foramen with erosion of the L5 body without injuring its stability. Besides, displacement of the right psoas muscle was also noted.

Electroneurography and electromyography at the lower extremities showed neurogenic damage with denervation signs of the right L5 nerve.

We decided to perform a one-step combined approach with the vascular surgeon because the lesion was too huge to allow a complete resection via a posterior approach and furthermore its tight relationship with the psoas muscle and the iliac vessels in the retroperitoneal space should be more safely managed via a retroperitoneal approach.

First we dissected free the nerve root from the tumor's capsule debulking the lesion and decompressing the spinal canal and then removed the residual portion after the control of the psoas muscle and the iliac vessels.

During the first step, we performed an L5-S1 hemilaminectomy, a facetectomy, and a resection of L5 transverse process. The tumor clearly originated from the L5 nerve root and invaded the vertebral foramen. We debulked the lesion using the ultrasonic surgical aspirator clearly maintaining the capsular surface to keep the correct margins of resection allowing a complete removal of the tumor.

Such approach allowed us not to sacrifice completely the nerve root, and after debulking, the mass was removed from the vertebral foramen up to the psoas muscle and the bony resection was minimal.

Immediately after the neurosurgical procedure, the vascular surgeon excised the residual retroperitoneal portion of the tumor by a right semilunar, subumbilical approach. He exposed the common iliac artery and the common iliac vein as well as the major part of the external iliac vein and of the hypogastric vein. The distal segment of the inferior vena cava and the aorta were also accurately visualized. Resection of the lesion medially to the psoas muscle was impossible because of its firm adherence to the muscle itself and the risk of vascular injury.

**Figure 3:**
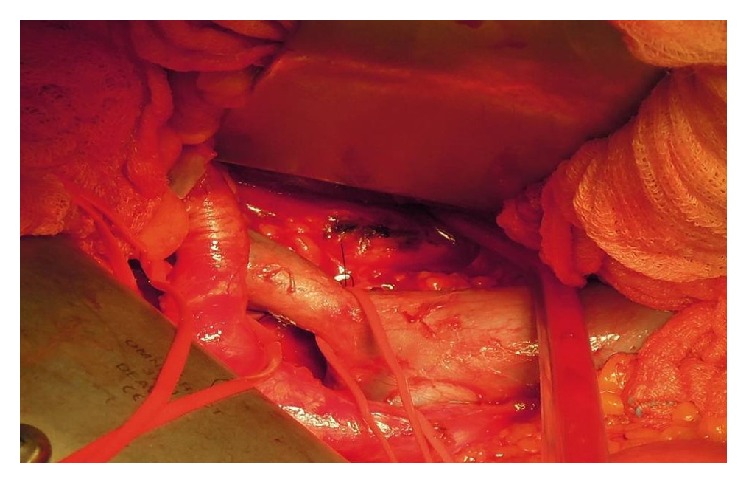
Intraoperative exposure of the tumor. The mass was firmly impinged to the bone and to the posterior aspect of the common iliac vein in its passage to extern iliac vein. In order not to damage the iliac veins and to save the hypogastric vein, the last lumbar vein was tied and the vena cava was raised and moved laterally.

The mass was firmly impinged to the bone and to the posterior aspect of the common iliac vein in its passage to extern iliac vein (Figure 3).

**Figure 4:**
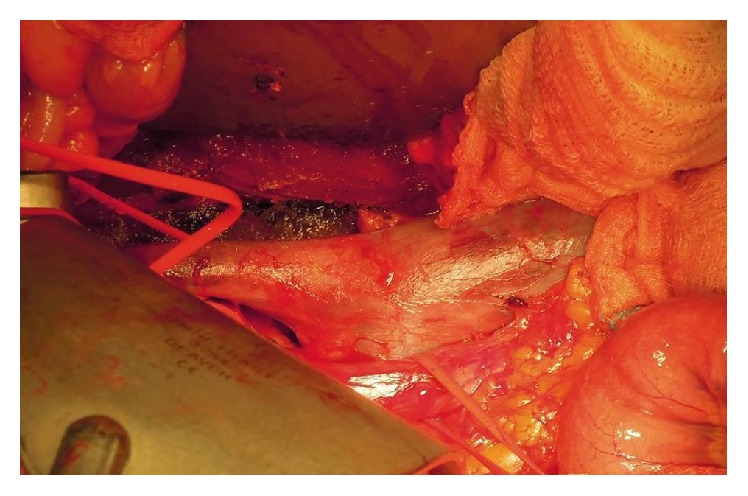
Intraoperative exposure of the tumor. The iliac vein was pulled upwards and medially with a widening of the operative field and the mass was finally removed, without traction, laterally. The haemostasis was satisfactory.

In order not to damage the iliac veins and to save the hypogastric vein, the last lumbar vein was tied and the vena cava was raised and moved laterally. The iliac vein was pulled upwards and medially with a widening of the operative field and the mass was finally removed, without traction, laterally (Figure 4).

The spine was considered stable and no additional fixation was performed because of a standard unilateral posterior approach and of the small portions of eroded vertebral body.

**Figure 5:**
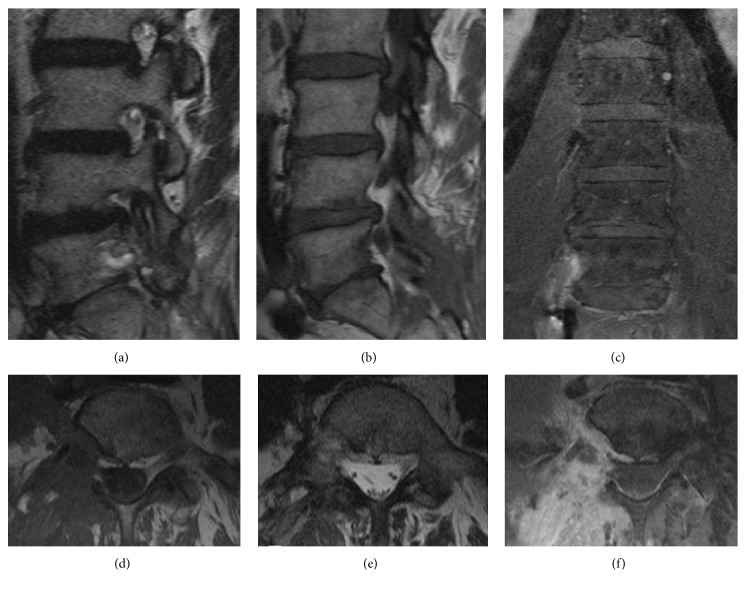
Postoperative spinal MRI. The mass was removed from the vertebral foramen up to the psoas muscle (a, b, and d). Resection of the lesion medially to the psoas muscle was achieved despite its firm adherence to the muscle itself and the risk of vascular injury (c, e, and f).

Five days after the operation, the patient was discharged and neurological examination revealed mild numbness in the right L5 dermatome that recovered 3 months later. There were no walking difficulties or back pain. The postoperative magnetic resonance image (MRI) confirmed (Figure 5) the complete resection of the tumor and a dynamic X-ray exam demonstrated the stability of the spine.

Clinical follow-up at 3, 6, and 12 months demonstrated the complete recovery of the patient.

Microscope examination revealed a mesenchymal spindle-cells tumor with rare mitotic activity. Tumor cells strongly and diffusely express S-100 protein, providing the diagnosis of benign schwannoma (WHO I).

## 3. Discussion

Giant schwannomas are very rare accounting for 0,3–5% of all these kind of tumors and affecting particularly the retroperitoneal space [1, 2].

Giant lumbar schwannomas eroding the vertebral body and extending into the retroperitoneal space are also rarer and in literature we found only 10 cases [1–10] representing a specific entity due to its rarity and complex surgical approach.

We focused our attention on the review of the cases of lumbar schwannomas eroding the vertebral bodies and invading the retroperitoneal space to evaluate the different surgical strategies and the considerations leading to 1 or 2 steps approaches.

The schwannoma is a typical benign tumor, well-encapsulated and without adherence with the surrounding tissue often presenting a delayed diagnosis due to their insidious behavior and slow growing, but large lesions present a more aggressive behavior with local compression, adherence, and bone rearrangement [1, 2].

Large retroperitoneal schwannomas often demonstrate such elements with important relationship with the large abdominal vessels accounting for high risk of intraoperative venous injury so that, in our opinion, involving vascular surgeon in the surgical team is strongly recommended.

However, such giant retroperitoneal lesions could mimic other aggressive intraabdominal tumors and osteoblastoma, chondrosarcoma, aneurismal bone cyst, or giant cell tumors must be considered in differential diagnosis.

Rapid growth, bone erosion, and invasion of the surrounding structures are similar for high grade sarcomas that, in doubtful cases, must be excluded preoperatively to choose the optimal strategy to avoid recurrence and metastases.

Invasion of the retroperitoneal space with bone erosion and myofascial plane extension defines a malignant schwannoma [2] and some authors report the necessity of a preoperative biopsy [2] being fundamental to choose an “en bloc” or a piecemeal resection [12].

Preoperative biopsy should be reserved to the quite doubtful cases because the cellular pleomorphism of the degenerated areas could produce the misdiagnosis of malignancy [10] and Sakalauskaite et al. [2] report a series of 25 retroperitoneal schwannomas in which only 7 among 25 cases obtained a preoperative diagnosis by biopsy.

Schwannoma demonstrates some radiological features suggesting the appropriate diagnosis as well circumscribed mass with heterogeneous contrast enhancement, calcifications, and cystic portions up to 40% in large tumor [2, 10].

MRI represents the exam of choice for preoperative diagnosis demonstrating hypointensity in T1 weighted images and hyperintensity in T2 weighted images [2]; during fat suppression sequence, the schwannoma maintains its hyperintensity [2].

After administration of contrast medium, schwannoma shows wide enhancement.

Angio-CT and abdominal CT study are absolutely mandatory in order to clarify the relationships of the tumor with the aorta, vena cava, and retroperitoneal structures.

The goal of surgery of these lesions is the complete removal preserving neural and intra-abdominal structures avoiding recurrence and postoperative adjuvant therapy [2] but literature reports very few papers about large retroperitoneal schwannoma eroding a lumbar vertebra and moreover about their treatment of choice relating to surgical strategy [1–10] (Table 1).

In 1971, Dickson et al. [6] reported a case of a 51-year-old female affected by a large schwannoma invading the L3 vertebral body; the patient, after a negative biopsy, underwent a partial posterior decompression after which she refused further surgery. Six months later, the patient completed the resection via a transabdominal approach and the vertebral defect was reconstructed with an iliac crest graft without postoperative complications.

In 1997, Kádár et al. [7] reported a case of giant schwannoma with vertebral erosion of L3; the authors approached such lesion in one sitting first via posterior laminectomy and canal decompression and then via a retroperitoneal approach. The resection was performed en bloc and followed by stabilization.

Napolitano et al. [8] described in 1998 a case of retroperitoneal schwannoma eroding the L4 body approached anteriorly, after a preoperative diagnostic biopsy, and followed by stabilization of the lumbar segment with an autograft, but unfortunately they did not report so descriptive data about their surgical strategy. The authors [8] well described the tenacious adherences with the vena cava and ureter.

In 1998, Chang et al. [3] described a case of intraosseous schwannoma involving the L4 vertebral body and, after a needle biopsy, removed it via a retroperitoneal approach followed by a lumbar stabilization. Their patient presented compression of thecal sac and a huge osteolytic lesion of the vertebral body and underwent a preoperative needle biopsy demonstrating a schwannoma. First the authors [3] performed tumor resection and anterior stabilization via an anterolateral retroperitoneal approach and 1 month later a posterior stabilization.

Paderni and Boriani [9] in 2002 described a giant retroperitoneal schwannoma eroding the L3 vertebral body and involving the right kidney, the ureter, the psoas muscle, and the vena cava. The authors [9], after a diagnostic preoperative biopsy, first approached it posteriorly with stabilization and detaching the tumor from the dura and then performed an en bloc resection of the tumor compressing the vena cava and the kidney via an anterior approach.

Case 3 of Daneshmand's series [10] in 2003 presented a giant L2 retroperitoneal schwannoma displacing aorta and vena cava, compressing the spinal canal, and destroying the vertebral bodies. Preoperative CT-guided biopsy reported the erroneous diagnosis of sarcoma and subsequently the patient underwent a thoracoabdominal approach followed by a lumbar corpectomy and fusion. The authors [10] chose a single step anterior approach and their postoperative course was uneventful nevertheless describing a difficult resection of the tumor from the spine.

Sofia et al. [11] in 2008 reported a 66-year-old female operated on for a large retroperitoneal schwannoma eroding the L2 left pedicle and vertebral body; their patient presented an involvement and a displacement of the kidney, the adrenal, and the aorta. The authors [11], after a positive biopsy, approached the lesion via a retroperitoneal route without a posterior step because of the stability of the spine and the lack of involvement of spinal canal; the patient was discharged after 10 days without general or neurological disturbances but a transient sensitive radicular deficit.

In 2008, Sakalauskaite et al. [2] reported a 56-year-old male affected by an 11 × 9 cm paravertebral tumor eroding L4 vertebral body; after a biopsy with the diagnosis of benign schwannoma, the patient underwent first a posterior approach followed 10 days later by a transabdominal approach.

In 2009, Chiang et al. [1] reported another case of giant retroperitoneal schwannoma (9 × 12 cm) eroding the L5 vertebral body, pedicle, and transverse process with an impingement of distal abdominal aorta and inferior cava vein and close relationship with common iliac vessels and ureter. The authors [1] after a positive biopsy planned a one-step surgery via a transretroperitoneal approach to obtain an en bloc resection and a final drilling of the involved bony structures that were reconstructed by a bony graft; they reported uneventful vascular damage well sutured by cardiovascular surgeon. No involvement of spinal canal was present. The only complaint they reported was a mild L5 weakness and dermatome numbness.

In 2009, Park et al. [4] report a 48-year-old female presenting with a completely eroded L4 vertebral body by a large schwannoma severely compressing the dural sac; the lesion was removed via a transretroperitoneal approach and the authors [4] first inserted an anterior cage after the corpectomy and then performed a posterior lumbar fusion to stabilize the spine.

The few papers in literature produce a lack of guidelines in treatment of such tumors so that surgical strategy must be discussed case by case relating to surgeon's experience and lesion's characteristics and many topics must be considered as the extension and the kind of the resection, the one-step or two-step approach, the kind of laparotomy, the involvement of abdominal structures and vessels, and the vertebral stability [2].

Usually, retroperitoneal location is correlated to lesion's large size (>3 cm) producing strong adherence with the surrounding tissue, vessels, and organs and behaving like a malignant schwannoma.

These lesions appear to be firm, adherent to surrounding structures, and often difficult to mobilize so we believe that a previous posterior approach can reduce their vascular source but furthermore produces an easier mobilization of the mass.

The cleavage and the disconnection of the tumor from its posterior vascular support and its radicular origin, achieving at the same time an optimal decompression of the spinal canal, dramatically produce an easier en bloc resection via the subsequent anterior approach.

Complete neurological deficit after complete sacrifice of the involved spinal root has been not so often reported with an incidence of about 23% [1] due to the functional compensation of the neighboring roots during the slow growth of the tumor.

Such kind of approach allows the surgeon to achieve a safer anterior en bloc resection because of the frequent involvement of abdominal structures as vena cava, aorta, iliac vessels, ureter, and kidney that could be easier managed after the reduction or the elimination of the posterior vascular supply and after the debridement of the lesion from the posterior neural structures.

We believe the posterior approach is also optimal to plan a vertebral stabilization when the stability of the spine is altered, so we advocate a 1-step surgery with an initial posterior approach followed by anterior removal of the lesion.

However, in literature, 6 cases [1, 3, 4, 8, 10, 11] among the 10 reported underwent first an anterior approach and only 2 of these underwent a following posterior approach.

In 4 of these, no spinal canal compression was reported and 3 authors performed only an anterior approach [1, 8, 11] while Park et al. [4] considered this approach to stabilize the spine inadequate and then performed a posterior stabilization.

The authors [1] report an incidental laceration of the common iliac vein due to its firm adhesion and impingement to the tumor repaired by a cardiovascular surgeon.

Case 3 of Daneshmand's series [6] suffered the important bias of the erroneous preoperative bioptic diagnosis of retroperitoneal sarcoma not expecting relationships with nerve roots or spinal canal.

The patient reported by Chang et al. [3], after an anterior retroperitoneal resection, had one month of absolute bed rest before undergoing a posterior stabilization.

We suggest a multidisciplinary approach involving two surgical teams (neurosurgeon, general/vascular surgeon) to complete the surgery in a single session with decompression of the spinal canal, disconnection of the tumor from its origin, and eventual stabilization when necessary.

This approach leads to an easier en bloc resection of the lesion via a subsequent retroperitoneal approach with important reduction of patient's affliction, medical expenses, and faster discharge.

Preoperative vascular study and a vascular surgeon being part of the surgical team is in our opinion mandatory because such lesions are often adherent to vital structures as vena cava and aorta; involvement of iliac and mesenteric vessels must be carefully considered.

Vascular identification and preparation of these vessels must be the first step of this surgery to prevent hemorrhage or acute bleeding.

Eventual spinal instability must be taken into account when a large vertebral amount must be resected and 8 on 10 patients of our review underwent spinal stabilization by anterior plating and grafting or posterior pedicle screws.

Two cases of 10 did not require a postoperative stabilization of the spine and according to these also our patient did not require stabilization.

Stabilization must be considered after wide bony resection or vertebral collapse and if postoperative dynamic X-rays films demonstrate instability.

## 4. Conclusion

We strongly suggest a 1-step surgery first approaching the dumbbell and the intraspinal schwannomas posteriorly achieving the decompression of the spinal canal and the cleavage of the tumor cutting the root of origin and the vascular supply and valuating the stability of the spine for potential artrodesis procedure. The patient must be then operated on via a retroperitoneal approach achieving the complete en bloc resection of the tumor. Only anterior approach could be evaluated for lesion not involving the spinal canal and not eroding a large amount of vertebral body not requiring a posterior stabilization.

## Figures and Tables

**Table 1:** Review of the literature.

Author	Year	Age/sex	Affected level	Biopsy	Procedure	Technique	Resection	Spinal fusion
Dickson et al. [6]	1971	51/F	L3	Yes	2 steps	Transperitoneal	Piecemeal	Yes
Kádár et al. [7]	1997	—	L3	No	1 step	Retroperitoneal	En bloc	Yes
Napolitano et al. [8]	1998	24/F	L4	Yes	1 step	Transperitoneal	—	Yes
Chang et al. [3]	1998	58/M	L4	Yes	2 steps	Retroperitoneal	Piecemeal	Yes
Paderni and Boriani [9]	2002	56/F	L3	Yes	1 step	Retroperitoneal	En bloc	Yes
Daneshmand et al. [10]	2003	46/F	L2	Yes	1 step	Transperitoneal	—	Yes
Sofia et al. [11]	2008	66/F	L2	Yes	1 step	Retroperitoneal	En bloc	No
Sakalauskaite et al. [2]	2008	56/M	L4	Yes	2 steps	Transperitoneal	Piecemeal	No
Chiang et al. [1]	2009	78/M	L5	Yes	1 step	Transperitoneal	En bloc	Yes
Park et al. [4]	2009	48/F	L4	No	1 step	Transperitoneal	—	Yes

## References

[1] Chiang E.-R., Chang M.-C., Chen T.-H. (2009). Giant retroperitoneal schwannoma from the fifth lumbar nerve root with vertebral body osteolysis: a case report and literature review. *Archives of Orthopaedic and Trauma Surgery*.

[2] Sakalauskaite M., Stanaitis J., Cepkus S., Pleckaitis M., Lunevicius R. (2008). Retroperitoneal giant schwannoma eroding lumbal vertebra: a case report with a literature review. *Central European Journal of Medicine*.

[3] Chang C. J., Huang J. S., Wang Y. C. (1998). Intraosseous Schwannoma of the fourth lumbar vertebra: case report. *Neurosurgery*.

[4] Park S.-C., Chung S.-K., Choe G., Kim H.-J. (2009). Spinal intraosseous schwannoma: a case report and review. *Journal of Korean Neurosurgical Society*.

[5] Li Q., Gao C., Juzi J. T., Hao X. (2007). Analysis of 82 cases of retroperitoneal schwannoma. *ANZ Journal of Surgery*.

[6] Dickson J. H., Thomas A., Waltz T. A. (1971). Intraosseous neurilemoma of the third lumbar vertebra. *The Journal of Bone and Joint Surgery*.

[7] Kádár E., Kisfaludi N., Czirják S., Jakab F. (1997). Resection of giant schwannoma by combined surgical exposure. *Acta Chirurgica Hungarica*.

[8] Napolitano C., Mutter D., Steib I. P., Christmann D., Marescaux J. (1998). A benign retroperitoneal schwannoma with vertebral lysis. A case report. *Annali Italiani di Chirurgia*.

[9] Paderni S., Boriani S. (2002). Schwannoma of L3. *La Chirurgia degli Organi di Movimento*.

[10] Daneshmand S., Youssefzadeh D., Chamie K. (2003). Benign retroperitoneal schwannoma: a case series and review of the literature. *Urology*.

[11] Sofia L., Currò G., Iapichino G., Melita G., Lorenzini C., Cucinotta E. (2008). Retroperitoneal giant schwannoma: a case report and review of the literature. *Chirurgia Italiana*.

[12] D'Andrea G., Caroli E., Capponi M. G. (2008). Retroperitoneal mesenchymal chondrosarcoma mimicking a large retroperitoneal sacral schwannoma. *Neurosurgical Review*.

